# Atherogenic index of plasma predicts cerebrovascular accident occurrence in antineutrophil cytoplasmic antibody-associated vasculitis

**DOI:** 10.1186/s12944-020-01360-1

**Published:** 2020-08-14

**Authors:** Sung Soo Ahn, Lucy Eunju Lee, Jung Yoon Pyo, Jason Jungsik Song, Yong-Beom Park, Sang-Won Lee

**Affiliations:** 1 Division of Rheumatology, Department of Internal Medicine, Yonsei University College of Medicine, 50-1 Yonsei-ro, Seodaemun-gu, Seoul, Republic of Korea 03722; 2 Institute for Immunology and Immunological Diseases, Yonsei University College of Medicine, Seoul, Republic of Korea

**Keywords:** Antineutrophil cytoplasmic antibody, Vasculitis, Atherogenic index of plasma, Cerebrovascular accident, Predictor

## Abstract

**Background:**

To investigate whether atherogenic index of plasma (AIP) at diagnosis is associated with the occurrence of cerebrovascular accident (CVA) or coronary artery disease (CAD) in antineutrophil cytoplasmic antibody-associated vasculitis (AAV).

**Methods:**

The medical records of 167 AAV patients on initial diagnosis was reviewed, and 300 healthy controls were included. AIP was calculated using the following equation: AIP = Log (triglyceride [mg/dL] / high-density lipoprotein cholesterol [mg/dL]). AAV patients were divided into two groups according to the AIP cut-off of 0.11. The event of stroke, transient ischemic attack, and cerebral hemorrhage was recorded as CVA, and CAD events consisted of either myocardial infarction and angina pectoris. CVA- and CAD- free survival rate between those with AIP ≥ 0.11 and < 0.11 were compared by the Kaplan-Meier analysis, and Cox hazard analysis was conducted to identify predictors of CVA.

**Results:**

The median age of AAV patients were 59.0 years, and 54 (32.3%) patients were male. One-hundred and fifteen (68.9%) patients had AIP < 0.11 and 52 (31.1%) had AIP ≥ 0.11. The mean Birmingham vasculitis activity score in AAV patients with AIP < 0.11 was lower than that seen in patients with AIP ≥ 0.11 (12.0 vs. 14.0, *P* = 0.041). AAV patients had a significantly higher AIP compared to controls (mean − 0.01 vs. -0.10, *P* < 0.001). During follow-up, the occurrence of CVA and CAD was observed in 16 (9.6%) and 14 (8.4%) patients, respectively. In Kaplan-Meier analysis, AAV patients with AIP ≥ 0.11 had significantly lower CVA-free survival rates than in those with AIP < 0.11 (*P* = 0.027), whereas there was no difference in CAD according to AIP (*P* = 0.390). Multivariable Cox analysis indicated that AIP ≥ 0.11 at diagnosis was the sole predictor of CVA (Hazard ratio 3.392, 95% confidence interval 1.076, 10.696, *P* = 0.037).

**Conclusions:**

AIP is significantly higher in AAV patients than in healthy controls, and AIP ≥ 0.11 at diagnosis is a significant predictor of CVA during follow-up. Stringent surveillance should be provided in AAV patients with AIP ≥ 0.11 regarding the occurrence of CVA.

**Trial registration:**

Retrospectively registered (4–2017-0673).

## Background

Antineutrophil cytoplasmic antibody (ANCA)-associated vasculitis (AAV) is a chronic inflammatory disorder (CID) that usually involves the small-sized vasculatures and has three distinct subtypes: microscopic polyangiitis (MPA), granulomatosis with polyangiitis, and eosinophilic granulomatosis with polyangiitis [1, 2]. Generally, AAV involves the vessels and induces necrotizing vasculitis within the arterioles, venules, and capillaries and could present with a wide spectrum of clinical manifestations [3]. Even though the pathogenesis of AAV is thought to be complex, it is now being increasingly understood that a breach of cellular and humoral immunity is responsible for the loss of self-tolerance, leading to inflammation and organ injury [4]. In addition, the overproduction of pro-inflammatory cytokines, such as tumor necrosis factor (TNF)-α, interferon-gamma, interleukin (IL)-1b, IL-6, IL-8, and IL-18, is responsible for amplifying the vicious loop [5].

Chronic inflammation is generally associated with deregulated lipid metabolism skewed towards an atherogenic profile, and it is typically characterized by an increase of triglyceride (TG) and decrease of high-density lipoprotein (HDL)-cholesterol [6, 7]. Even though multiple factors have been suggested regarding this phenomenon, the secretion of multiple inflammatory cytokines, i.e. TNF, IL-1, IL-2, and IL-6, is reported to be linked to the elevation of TG level, which is induced by accelerated lipolysis in the adipose tissue and the synthesis of fatty acids in the liver, while inhibiting hepatic fatty acid oxidation [8]. On the other hand, pro-inflammatory cytokines are also implicated in the decrease of HDL-cholesterol as a consequence of impaired production of apolipoprotein A-1, which is a major protein constituting the HDL-cholesterol [9]. Moreover, diminished formation of cholesterol ester, structural and functional alteration of HDL-cholesterol, and increased HDL-cholesterol clearance have been also thought be relevant to the decreased HDL-cholesterol level in the presence of chronic inflammation [7].

Emerging evidences now clearly indicate that patients with CID are prone to cardiovascular diseases (CVD) such as cerebrovascular accident (CVA), coronary artery disease (CAD), and thromboembolic diseases [10–12]. Notably, atherogenic index of plasma (AIP), which is calculated based on serum TG and HDL-cholesterol, is one of the laboratory indices indicating atherogenic status, and this has been used to assess the extent of dyslipidemia and predict the potential of developing CVA and CAD in various medical conditions [13–15]. Given that patients with CID are more often affected by CVDs than in healthy subjects and that increase of TG and decrease of HDL-cholesterol is present in those with chronic inflammation, it is possible that AIP is elevated in AAV patients and is associated with CVA and CAD. However, there have been no studies that determined the predictive potential of AIP regarding CVA and CAD events in AAV. Hence, this study investigated whether AIP in patients with AAV is higher than that in controls and to check whether this is associated the occurrence of CVA and CAD.

## Methods

### Patient inclusion

The medical records of 216 AAV patients when the initial diagnosis was made, were retrospectively reviewed. Patients were classified as AAV at the Division of Rheumatology in Severance Hospital, during the period from October 2000 to December 2019, based on the 2007 European Medicines Agency algorithm and the 2012 Chapel Hill Consensus Conferences definitions [1, 16]. The patients had well-documented medical records with accessible clinical and laboratory results, including Birmingham vasculitis activity score (BVAS) and five-factor score (FFS) [17, 18]. ANCA was detected using indirect immunofluorescence assay and antigen-specific enzyme-linked immunosorbent assays for myeloperoxidase and proteinase 3. When the patients did not have ANCAs detected, the diagnosis of ANCA-negative vasculitis was made based on the clinical features and/or the histologic findings. All study subjects were followed up for at least 3 months after the diagnosis of AAV. At the time of diagnosis, patients were not on immunosuppressive agents and were not on medications to treat dyslipidemia. Further characteristics that would lead to a false-positive ANCA result, such as coexisting malignancies and serious infections, were also considered as exclusion criteria. Of the 216 AAV patients, 37 were excluded because TG and HDL-cholesterol levels were not assessed. Furthermore, 12 patients were currently taking drugs to treat dyslipidemia. Finally, 167 patients were included and analyzed in this study. In addition, body mass index (BMI) and lipid levels of TG, HDL-cholesterol, and low-density lipoprotein (LDL)-cholesterol from 300 age- and gender-matched healthy controls, who had visited the healthcare center in Severance Hospital for a routine health examination and were not on medications to treat dyslipidemia, were assessed. The Institutional Review Board of Severance Hospital approved this study and was performed according to the ethical guidelines set forth in the Declaration of Helsinki (4–2017-0673).

### Baseline data and the definition of clinical outcomes

Patients’ demographic data such as age, gender, BMI, smoking history, and AAV subtypes were collected. ANCA positivity as well as AAV-specific indices of BVAS and FFS were obtained. Clinical manifestations and the presence of comorbidities, such as chronic kidney disease (stage III–V), diabetes mellitus, hypertension, and interstitial lung disease, were also assessed. In addition, the laboratory results including acute phase reactants of erythrocyte sedimentation rate (ESR) and C-reactive protein (CRP) level, as well as AIP-related variables ─ serum total cholesterol, TG, HDL-cholesterol, and LDL-cholesterol levels ─ were reviewed. The clinical outcomes evaluated during the follow-up consisted of all-cause mortality, CVA, and CAD. In this study, we defined all-cause mortality as death regardless of the cause. We included events of cerebral infarction, transient ischemic attack, and cerebral hemorrhage as CVA and included acute coronary syndrome, including myocardial infarct and angina pectoris, as CAD. The follow-up duration was defined as the duration between the date of AAV diagnosis until the occurrence of clinical outcomes or the last visit, when subjects did not have an event corresponding to clinical outcomes.

### Calculation of AIP

AIP was calculated using the following equation: AIP = Log (TG (mg/dL) / HDL-cholesterol (mg/dL)), as described previously [13]. Based on the results from previous studies, patients could be divided into three categories based on their AIP value. An AIP < 0.11 as low risk, AIP of 0.11–0.21 as intermediate risk, and AIP > 0.21 as high risk [19, 20]. In this study, AAV patients were divided into two groups according to AIP as follows: AAV patients with AIP < 0.11 (*N* = 115) and AAV patients with AIP ≥ 0.11(*N* = 52).

### Statistical analyses

Continuous variables that were normally distributed are expressed as mean (standard deviation) and as median (interquartile range) when non-normally distributed. Categorical variables are expressed as number and the percentage. Significant differences between continuous variables were assessed using Student’s t-test, Mann-Whitney U test, and analysis of variance, whereas differences between the categorical variables were analyzed by the Chi-square and Fisher’s exact tests, as appropriate. Associations and the correlation coefficient between continuous variables were derived using the Pearson correlation analysis or Spearman correlation analysis. Comparison of the clinical outcome free survivals rates were analyzed using the Kaplan-Meier analysis with the log-rank test. Multivariable Cox hazards analysis was carried out using variables that showed statistical significance in the univariable analysis to identify predictors of CVA by the forward entry method. A *P*-value < 0.05 was considered statistically significant. All statistical analyses were conducted using SPSS software version 23 for Windows (IBM Corp., Armonk, NY, USA).

## Results

### Clinical characteristics of AAV patients at baseline

Of the included 167 patients, the median age of the patients was 59 years, 54 (32.3%) were male, and the mean BMI was 22.1 kg/m^2^, respectively. The most common AAV subtype in the patients was MPA (*N* = 92, 55.1%), and ANCA was detected in 133 patients (79.6%). The most common clinical features were renal (62.3%) and pulmonary (58.1%) manifestations; hypertension as a comorbid condition was most frequently (53.3%) observed. The laboratory test results are shown in Table 1. The mean calculated AIP was − 0.01, and 115 (68.9%) and 52 (31.1%) patients were included in the AIP < 0.11 and AIP ≥ 0.11 group, respectively. Patients with AIP ≥ 0.11 had a higher BVAS compared to those with AIP < 0.11 (*P* = 0.041), even though there were no difference in FFS (*P* = 0.656). However, total serum protein level was significantly lower in patients with AIP ≥ 0.11 than in those with AIP < 0.11 (6.7 mg/dL vs. 6.4 mg/dL, *P* = 0.043). As for AIP-related variables, TG level was higher in the AIP ≥ 0.11 group than in the AIP < 0.11 group, while the level of HDL-cholesterol was lower (both *P* < 0.001) (Table 1).

**Table 1:** Clinical characteristics of AAV patients at diagnosis

Variables	All AAV patients (***N*** = 167)	AAV patients with AIP < 0.11 (***N*** = 115)	AAV patients with AIP ≥ 0.11 (***N*** = 52)	***P***-value
**Demographic data**				
Age (years)	59.0 (22.0)	60.0 (24.0)	56.0 (19.8)^a^	0.369
Male gender (N, (%))	54 (32.3)	34 (29.6)	20 (38.5)	0.255
Body mass index (kg/m^2^)	22.1 (4.5)^a^	22.0 (4.8)^a^	23.2 (4.3)^a^	0.324
Smoking history (N, (%))	6 (3.6)	3 (2.6)	3 (5.8)	0.310
**AAV Subtypes (N, (%))**				0.641
MPA	92 (55.1)	64 (55.7)	28 (53.8)	
GPA	38 (22.8)	24 (20.9)	14 (26.9)	
EGPA	37 (22.2)	27 (23.5)	10 (19.2)	
**ANCA positivity (N, (%))**				
MPO-ANCA (or P-ANCA) positivity	114 (68.3)	79 (68.7)	35 (67.3)	0.858
PR3-ANCA (or C-ANCA) positivity	27 (16.2)	16 (13.9)	11 (21.2)	0.239
Both ANCA positivity	8 (4.8)	6 (5.2)	2 (3.8)	1.000
ANCA negativity	34 (20.4)	26 (22.6)	8 (15.4)	0.283
**AAV-specific indices**				
BVAS	12.0 (11.0)^a^	12.0 (11.0)^a^	14.0 (10.0)^a^	0.041
FFS	1.0 (1.0)	1.0 (1.0)	1.0 (1.0)	0.656
**Clinical manifestations (N, (%))**				
General	75 (44.9)	48 (41.7)	27 (51.9)	0.221
Cutaneous	38 (22.8)	29 (25.2)	9 (17.3)	0.259
Muco-membranous /Ocular	10 (6.0)	5 (4.3)	5 (9.6)	0.184
Ear nose throat	75 (44.9)	51 (44.3)	24 (46.2)	0.828
Pulmonary	97 (58.1)	69 (60.0)	28 (53.8)	0.455
Cardiovascular	43 (25.7)	27 (23.5)	16 (30.8)	0.318
Gastrointestinal	9 (5.4)	7 (6.1)	2 (3.8)	0.553
Renal	104 (62.3)	69 (60.0)	35 (67.3)	0.367
Nervous	56 (33.5)	38 (33.0)	18 (34.6)	0.842
**Comorbidities (N, (%))**				
Chronic kidney disease (stage 3–5)	53 (31.7)	33 (28.7)	20 (38.5)	0.209
Diabetes mellitus	51 (30.5)	35 (30.4)	16 (30.8)	0.965
Hypertension	89 (53.3)	57 (49.6)	32 (61.5)	0.151
Interstitial lung disease	50 (29.9)	36 (31.3)	14 (26.9)	0.567
**Laboratory results**				
White blood cell count (/mm^3^)	9210.0 (6347.5)	9180.0 (6430.0)	10,230.0 (6000.0)	0.927
Hemoglobin (g/dL)	11.3 (2.3)^a^	11.4 (2.3)^a^	11.0 (2.5)^a^	0.235
Platelet count (× 1000/mm^3^)	308.0 (166.0)	311.0 (158.5)	294.0 (195.0)	0.949
Fasting glucose (mg/dL)	104.0 (36.0)	103.0 (34.0)	106.0 (38.0)	0.466
BUN (mg/dL)	18.4 (22.4)	18.0 (23.0)	24.2 (21.6)	0.078
Creatinine (mg/dL)	1.0 (1.3)	0.9 (1.0)	1.3 (2.0)	0.105
Total serum protein (g/dL)	6.6 (1.3)	6.7 (1.1)	6.4 (1.0)^a^	0.043
Serum albumin (g/dL)	3.5 (0.8)^a^	3.5 (0.7)^a^	3.3 (0.8)^a^	0.068
ALP (IU/L)	72.0 (38.0)	69.0 (36.0)	77.0 (51.0)	0.339
AST (IU/L)	18.5 (9.0)	19.0 (8.0)	17.0 (10.0)	0.121
ALT (IU/L)	16.5 (14.8)	15.0 (13.5)	19.0 (18.0)	0.985
Total bilirubin (mg/dL)	0.5 (0.3)	0.5 (0.3)	0.5 (0.4)	0.620
ESR (mm/hr)	64.0 (69.3)	64.0 (67.0)	66.8 (39.6)^a^	0.406
CRP (mg/L)	17.0 (85.8)	15.0 (69.3)	25.0 (119.3)	0.123
**AIP-related variables**				
Total cholesterol (mg/dL)	178.0 (58.0)	182.1 (45.8)^a^	187.0 (58.1)^a^	0.561
TG (mg/dL)	113.0 (73.0)	97.5 (35.5)^a^	174.5 (67.8)	< 0.001
HDL-cholesterol (mg/dL)	51.0 (23.0)	55.0 (25.0)	41.4 (14.6)^a^	< 0.001
LDL-cholesterol (mg/dL)	104.6 (41.6)	106.0 (33.1)^a^	102.2 (45.1)	0.917
AIP	−0.01 (0.2)^a^	− 0.1 (0.3)	0.3 (0.1)^a^	< 0.001

Values are expressed as a median (interquartile range) or N (%)

*AAV* ANCA-associated vasculitis, *ANCA* Antineutrophil cytoplasmic antibody, *AIP* Atherogenic index of plasma, *MPA* Microscopic polyangiitis, *GPA* Granulomatosis with polyangiitis, *EGPA* Eosinophilic GPA, *MPO* Myeloperoxidase, *P* Perinuclear, *PR3* Proteinase 3, *C* Cytoplasmic, *BVAS* Birmingham vasculitis activity score, *FFS* Five-factor score, *BUN* Blood urea nitrogen, *ALP* Alkaline phosphatase, *AST* Aspartate aminotransferase, *ALT* Alanine aminotransferase, *ESR* Erythrocyte sedimentation rate, *CRP* C-reactive protein, *TG* Triglyceride, *HDL* High-density lipoprotein, *LDL* Low-density lipoprotein

^a^ Normally distributed data are expressed as mean (standard deviation)

On comparing demographic and AIP-related variables with healthy controls, AAV patients were found to have a significantly lower BMI (*P* < 0.001) and higher TG and HDL-cholesterol levels (median 113.0 mg/dL vs. 94.0 mg/dL, *P* = 0.002 and median 51.0 mg/dL vs. 50.0 mg/dL, *P* = 0.016). Moreover, AAV patients exhibited a significantly higher AIP value than controls (mean − 0.01 vs. -0.10, *P* < 0.001) (Table 2).

**Table 2:** Comparison of demographic data and AIP-related variables between AAV patients and controls

Variables	AAV patients (***N*** = 167)	Controls (***N*** = 300)	***P***-value
**Demographic data**			
Age (years)	59.0 (22.0)	58.0 (21.0)	0.382
Male gender (N, (%))	54 (32.3)	82 (27.3)	0.254
Body mass index (kg/m^2^)	22.3 (3.1)^a^	23.3 (3.4)	< 0.001
**AIP-related variables**			
TG (mg/dL)	113.0 (73.0)	94.0 (60.0)	0.002
HDL-cholesterol (mg/dL)	51.0 (23.0)	50.0 (19.0)	0.016
LDL-cholesterol (mg/dL)	104.6 (41.6)	106.0 (48.0)	0.211
AIP	−0.01 (0.2)^a^	−0.10 (0.2)^a^	< 0.001

Values are expressed as a median (interquartile range) or N (%)

*AIP* Atherogenic index of plasma, *AAV* ANCA-associated vasculitis, *ANCA* Antineutrophil cytoplasmic antibody, *TG* Triglyceride, *HDL* High-density lipoprotein, *LDL* Low-density lipoprotein

^a^ Normally distributed data are expressed as mean (standard deviation)

### Correlation of AIP with continuous variables

At AAV diagnosis, AIP was found to be positively correlated with ESR (*r* = 0.171, *P* = 0.028), CRP (*r* = 0.169, *P* = 0.030), and blood urea nitrogen (*r* = 0.187, *P* = 0.016) and negatively correlated with total serum protein and serum albumin (*r* = − 0.201, *P* = 0.010 and *r* = − 0.209, *P* = 0.007). In addition, AIP was highly correlated with TG and HDL-cholesterol (*r* = 0.770, *P* < 0.001 and *r* = − 0.579, *P* < 0.001). AIP was not significantly correlated with age, BMI, BVAS, FFS, and the remaining laboratory variables (Table 3).

**Table 3:** Relationship between AIP and continuous variables at diagnosis

Variables	r-value	***P***-value
Age	0.068	0.386
Body mass index^a^	0.102	0.191
White blood cell count (/mm^3^)	0.080	0.309
Hemoglobin (g/dL)^a^	−0.124	0.113
Platelet count (× 1000/mm^3^)	0.069	0.377
Fasting glucose (mg/dL)	0.117	0.133
BUN (mg/dL)	0.187	0.016
Creatinine (mg/dL)	0.141	0.071
Total serum protein (g/dL)	−0.201	0.010
Serum albumin (g/dL)^a^	−0.209	0.007
ALP (IU/L)	0.100	0.199
AST (IU/L)	−0.093	0.237
ALT (IU/L)	−0.036	0.647
Total bilirubin (mg/dL)	0.045	0.569
ESR (mm/hr)	0.171	0.028
CRP (mg/L)	0.169	0.030
Total cholesterol (mg/dL)	−0.075	0.338
TG (mg/dL)	0.770	< 0.001
HDL-cholesterol (mg/dL)	−0.579	< 0.001
LDL-cholesterol (mg/dL)	−0.035	0.650
BVAS^a^	0.124	0.111
FFS	0.093	0.232

*AIP* Atherogenic index of plasma, *BUN* Blood urea nitrogen, *ALP* Alkaline phosphatase, *AST* Aspartate aminotransferase, *ALT* Alanine aminotransferase, *ESR* Erythrocyte sedimentation rate, *CRP* C-reactive protein, *TG* Triglyceride, *HDL* High-density lipoprotein, *LDL* Low-density lipoprotein, *BVAS* Birmingham vasculitis activity score, *FFS* Five-factor score

^a^ Normally distributed data

### Clinical outcomes and medications during follow-up

During the follow-up period, 18 patients died, and 16 and 14 patients experienced CVA and CAD events, respectively. Concerning the medications used to treat AAV, glucocorticoids were most frequently administered (*N* = 155, 92.8%), followed by cyclophosphamide (*N* = 87, 52.1%) and azathioprine (*N* = 82, 49.1%) (Table 4).

**Table 4:** Outcomes and the medications administered in AAV patients during follow-up

AAV patients	Values
**Clinical outcomes during follow-up (N, (%))**	
All-cause mortality (N, (%))	18 (10.8)
Follow-up duration based on all-cause mortality (months)	33.7 (65.6)
CVA (N, (%))	16 (9.6)
Follow-up duration based on CVA (months)	30.5 (64.0)
CVD (N, (%))	14 (8.4)
Follow-up duration based on CVD (months)	32.8 (63.6)
**Medications administered during follow-up (N, (%))**	
Glucocorticoid	155 (92.8)
Cyclophosphamide	87 (52.1)
Rituximab	29 (17.4)
Azathioprine	82 (49.1)
Mycophenolate mofetil	22 (13.2)
Tacrolimus	11 (6.6)
Methotrexate	12 (7.2)

Values are expressed as a median (interquartile range, IQR) or N (%)

*AAV* ANCA-associated vasculitis, *ANCA* Antineutrophil cytoplasmic antibody, *CVA* Cerebrovascular accident, *CVD* Cardiovascular disease

### Comparison of clinical outcomes between AAV patients with AIP < 0.11 and those with AIP ≥ 0.11

**Fig. 1:**
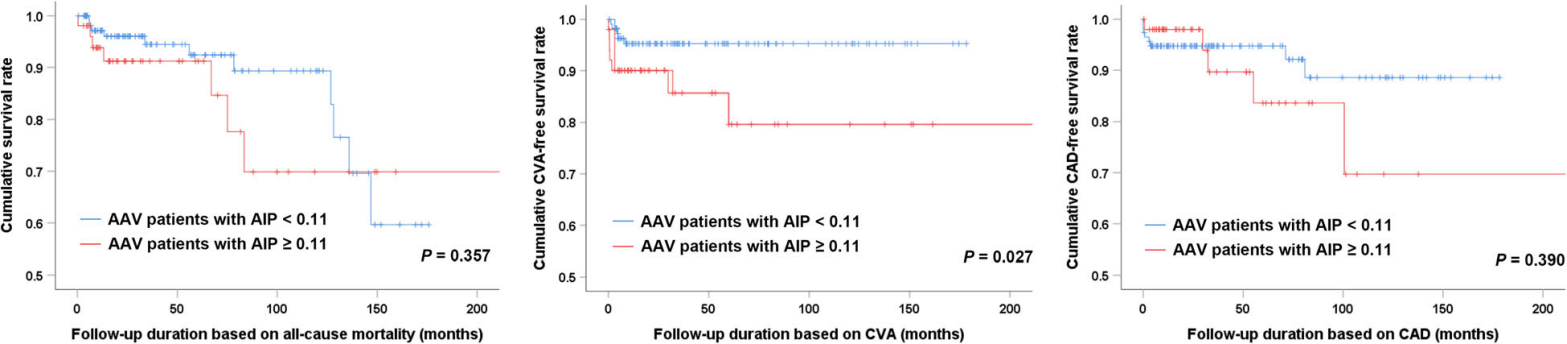
Comparison of the cumulative clinical outcome free survival rate in patients with AIP < 0.11 and AIP ≥ 0.11. Among the clinical outcomes, AAV patients with AIP ≥ 0.11 exhibited a significantly lower cumulative CVA-free survival rate than those with AIP < 0.11, while there was no difference regarding all-cause mortality and CAD. AIP: atherogenic index of plasma; AAV: ANCA-associated vasculitis; ANCA: antineutrophil cytoplasmic antibody; CVA: cerebrovascular accident; CAD: coronary artery disease

Regarding clinical outcomes, AAV patients with AIP ≥ 0.11 exhibited a significantly lower CVA-free survival rate than those with AIP < 0.11 (*P* = 0.027). However, there were no significant differences regarding all-cause mortality and CAD between patients with AIP < 0.11 and AIP ≥ 0.11 (*P* = 0.357 and *P* = 0.390) (Fig. 1).

### Cox hazards analysis for the prediction of CVA

To investigate whether AIP ≥ 0.11 at diagnosis could independently predict CVA occurrence during follow-up, we compared the predictive potential of conventional risk factors for CVA, ANCA types, AAV-specific indices, acute phase reactants, and AIP using the Cox hazards analysis. In the univariable analysis, both AIP ≥ 0.11 (Hazard ratio (HR) 3.391, 95% confidence interval (CI) 1.075, 10.695, *P* = 0.037) and CRP (HR 1.008, 95% CI 1.001, 1.016, *P* = 0.035) were significantly associated with CVA during follow-up. In the multivariable analysis, only AIP ≥ 0.11 at diagnosis was revealed to be significantly associated with CVA during follow-up (HR 3.392, 95% CI 1.076, 10.696, *P* = 0.037) (Table 5).

**Table 5:** Predictors for the occurrence of CVA during follow-up

Variables	Univariable			Multivariable		
	HR	95% CI	***P***-value	HR	95% CI	***P***-value
**Demographic data**						
Age (years)	1.027	0.982, 1.074	0.243			
Male gender	0.862	0.259, 2.874	0.809			
Body mass index (kg/m^2^)	0.991	0.826, 1.190	0.924			
Smoking history	0.047	0.000, 32,787.590	0.656			
**Comorbidities**						
Chronic kidney disease (stage 3–5)	1.069	0.322, 3.551	0.914			
Diabetes mellitus	1.527	0.484, 4.810	0.470			
Hypertension	1.636	0.492, 5.442	0.422			
**ANCA positivity**						
MPO-ANCA (or P-ANCA) positivity	0.678	0.215, 2.142	0.508			
PR3-ANCA (or C-ANCA) positivity	1.041	0.228, 4.760	0.958			
**AAV-specific indices**						
BVAS	1.074	0.995, 1.160	0.066			
FFS	1.476	0.851, 2.559	0.165			
**Acute phase reactants**						
ESR (mm/hr)	1.001	0.986, 1.017	0.860			
CRP (mg/L)	1.008	1.001, 1.016	0.035			
**AIP ≥ 0.11**	3.391	1.075, 10.695	0.037	3.392	1.076, 10.696	0.037

*CVA* Cerebrovascular accident, *ANCA* Antineutrophil cytoplasmic antibody, *MPO* Myeloperoxidase, *P* Perinuclear, *PR3* Proteinase 3, *C* Cytoplasmic, *BVAS* Birmingham vasculitis activity score, *FFS* Five factor score, *ESR* Erythrocyte sedimentation rate, *CRP* C-reactive protein, *AIP* Atherogenic index of plasma

### Comparison of AIP according to gender, body mass index, age, and AAV subtypes

**Fig. 2:**
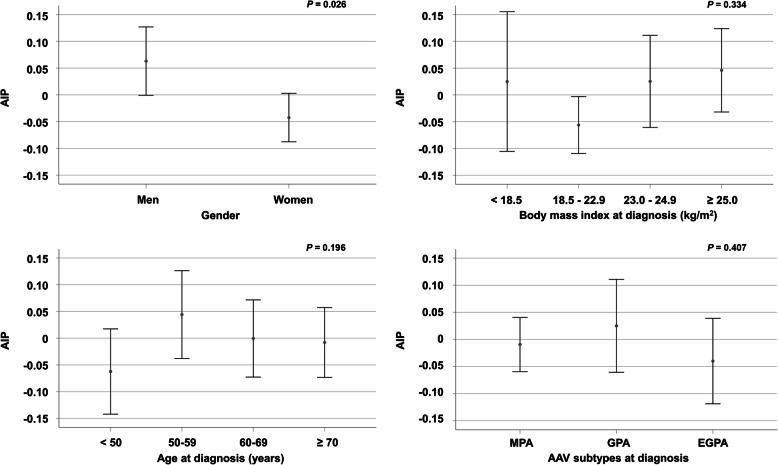
Comparison of AIP based on gender, body mass index, age, and AAV subtypes. Male patients with AAV exhibited a higher AIP than female patients with AAV. However, no significant differences in AIP according to body mass index, age, and AAV subtypes were found. AIP: atherogenic index of plasma; AAV: ANCA-associated vasculitis; ANCA: antineutrophil cytoplasmic antibody; MPA: microscopic polyangiitis; GPA: granulomatosis with polyangiitis; EGPA: eosinophilic GPA

To exclude the possibility of the influence of gender, BMI, age, and AAV subtypes in AIP, a subgroup analysis was performed. Male patients with AAV had significantly higher AIP than female patients with AAV (*P* = 0.026). However, there were no significant differences in AIP regarding BMI (divided based on the Asian Pacific cut-off values) (*P* = 0.334) and age (*P* = 0.196) [21]. Furthermore, AIP was not found to differ based on AAV subtypes (*P* = 0.407) (Fig. 2).

## Discussion

In line with the knowledge that patients with CIDs are at increased risk of developing CVDs, several studies have demonstrated that the risk of CVDs is heightened in AAV patients. A long-term population-based study revealed that the risk of CVD and CVA in AAV patients were 3- and 8-fold higher than those in matched subjects [22]. In addition, a retrospective study that was performed in the United Kingdom has shown that AAV patients have high incidence of arterial and venous thrombosis [23]. Moreover, a meta-analysis by Houben et al. demonstrated that the risk of developing cardiovascular events was higher in AAV patients compared to the general population [24]. Therefore, it is clinically important to uncover predictors that could help estimate the development of CVDs in AAV patients, as this could be a potentially life-threatening event.

To the best of our knowledge, this is the first study that evaluated the clinical effectiveness of using AIP to predict CVA and CVD in AAV patients. Consistent with what was initially expected, the observations from this study demonstrated that AAV patients had significantly higher AIP compared to healthy controls (0.01 vs. -0.12, *P* < 0.001). Moreover, AAV patients with AIP ≥ 0.11 on diagnosis exhibited significantly higher disease activity at baseline, and the occurrence of CVA events during the follow-up was more frequent compared to those with AIP < 0.11. In addition, Cox hazards analysis revealed that AIP ≥ 0.11 at diagnosis is an independent predictor for CVA during follow-up, even when various conventional risk factors for CVA, ANCA types, AAV-specific indices, and acute phase reactants were taken into consideration [17, 18, 25–28]. On the basis of the results of this study, it could be suggested that the occurrence of CVA should be actively monitored in AAV patients, especially in those with AIP ≥ 0.11, when the initial diagnosis is established.

**Fig. 3:**
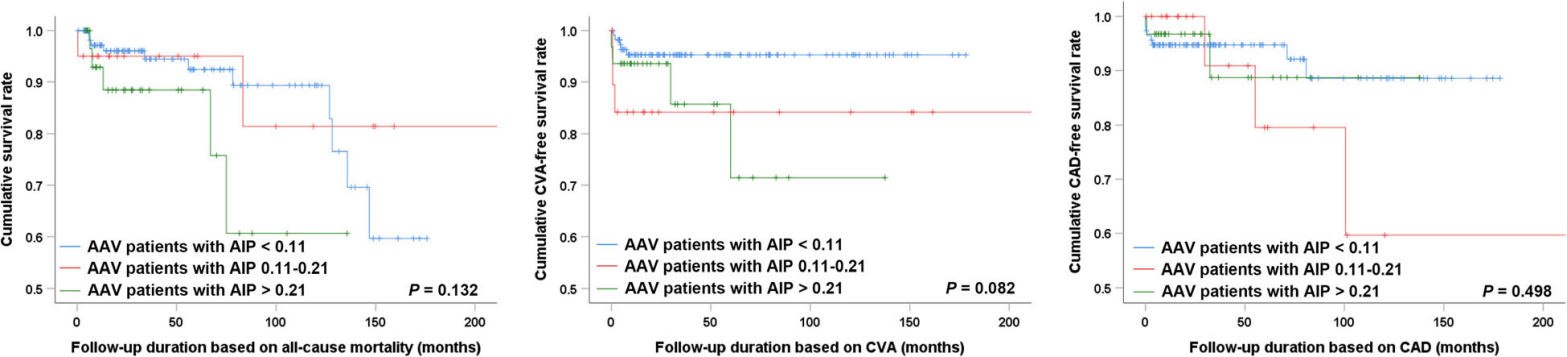
Comparison of the cumulative clinical outcome free survival rate based on the different cut-off of AIP. The frequency of CVA seemed to be higher in patients with AIP 0.11–0.21 and AIP ≥ 0.21 compared to those with AIP < 0.11, although statistical significance has not been reached. CVA: cerebrovascular accident; AIP: atherogenic index of plasma; CAD: coronary artery disease

In the present study, the cut-off value of AIP ≥ 0.11 was adopted to predict de novo CVA events in AAV patients; this was done since the number of patients with AIP 0.11–0.21 (*N* = 20) and AIP > 0.21 was small (*N* = 32) and since the events of CVA and CAD were observed in a relatively small number of patients. Therefore, categorizing patients into the three different groups of AIP < 0.11, AIP 0.11–0.21, and AIP > 0.21 could result in a false negative result owing to a low statistical power. Indeed, when the clinical outcomes were compared by dividing the patients into three groups, the patients with AIP < 0.11 were less likely to experience CVA compared to those with AIP 0.11–0.21 and AIP > 0.21, even though the risk of developing CVA was not directly incremental (Fig. 3).

Previous studies have demonstrated a variable range of AIPs, and variations could arise because of differences in ethnicity, gender, geographical factors, and the medical conditions investigated. A study by Zhu et al. has shown that the mean AIP values in Chinese people with and without obesity were 0.13 and − 0.04 [29]. On the other hand, Wu et al. identified the mean value of AIP in postmenopausal women with CAD and without CAD as 0.20 and 0.10, respectively; furthermore, a study that estimated AIP in subjects undergoing chronic dialysis reported median values of AIP as 0.47 [13, 30]. Notably, several studies have been performed to evaluate AIP levels in CIDs, and a study that compared AIP levels in patients with SLE and controls showed that SLE patients had significantly higher AIP levels than controls, which is consistent with the result of this study [31]. Moreover, it was also described that AIP could be a predictor of subclinical atherosclerosis, particularly carotid artery intima-media thickness, in subjects with SLE, Behçet disease, and ankylosing spondylitis, emphasizing that subjects with higher AIP could be more vulnerable to CVA among patients with CIDs [31–33]. However, as most of the studies that were performed in patients with CIDs did not directly evaluate the incidence of CVAs, additional research is necessary to determine the relationship between AIP and CVAs in CIDs.

Besides AIP, previous data have proposed that higher cholesterol levels, such as TC, LDL-cholesterol, and TG could be used to predict the incidence of CVDs, even though inconsistent results were obtained across studies [34–37]. Accordingly, when the predictive value of different laboratory measures comprising AIP was investigated, AIP ≥ 0.11 and HDL-cholesterol were found to be significantly associated with the incidence of CVA. However, given that AIP and HDL-cholesterol are closely associated because AIP level also includes the level of HDL-cholesterol in its calculation, further research is necessary to identify which is the most appropriate measure in estimating CVAs in AAV.

It has been acknowledged that higher disease activity is generally associated with increased risk of CVDs in patients with CIDs [38, 39]. Accordingly, on comparing the baseline characteristics of patients with AIP ≥ 0.11 and AIP < 0.11, it was found that the difference of BVAS between the groups was significant (*P* = 0.041). Furthermore, in regard to the medications administered during follow-up, both cyclophosphamide (63.5% vs. 47.0%, *P* = 0.048) and rituximab (26.9% vs. 13.0%, *P* = 0.028) were administered more commonly to AAV patients with AIP ≥ 0.11 than to those with AIP < 0.11 (Table 6). Based on the recommendations for the management of AAV, either cyclophosphamide or rituximab together with glucocorticoid should be given to AAV patients with life-threatening disease [40]. Taken together, it can be speculated that the inflammatory burden in AAV patients with AIP ≥ 0.11 is significantly higher than that of patients with AIP < 0.11 during follow-up, leading to an increased risk for CVA. In particular, it should be noted that the sum of the inflammatory burdens could not be simply estimated through BVAS, which consists of multiple measures that may not properly indicate the dynamic changes of vascular inflammation [41].

**Table 6:** Comparison of medication usage between patients with AIP ≥ 0.11 and AIP < 0.11 during follow-up

	All AAV patients (***N*** = 167)	AAV patients with AIP < 0.11 (***N*** = 115)	AAV patients with AIP ≥ 0.11 (***N*** = 52)	***P***-value
**Medications administered during follow-up (N, (%))**				
Glucocorticoid	155 (92.8)	104 (90.4)	51 (98.1)	0.107
Cyclophosphamide	87 (52.1)	54 (47.0)	33 (63.5)	0.048
Rituximab	29 (17.4)	15 (13.0)	14 (26.9)	0.028
Azathioprine	82 (49.1)	52 (45.2)	30 (57.7)	0.135
Mycophenolate mofetil	22 (13.2)	13 (11.3)	9 (17.3)	0.288
Tacrolimus	11 (6.6)	6 (5.2)	5 (9.6)	0.289
Methotrexate	12 (7.2)	10 (8.7)	2 (3.8)	0.345

*AIP* Atherogenic index of plasma, *AAV* ANCA-associated vasculitis, *ANCA* Antineutrophil cytoplasmic antibody

A previous study suggested that the major factors influencing AIP were gender, obesity, and older age [42]. Accordingly, in this study, a subgroup analysis was performed to determine whether these factors affected the level of AIP. Of note, even though it was also revealed that male AAV patients exhibited significantly elevated AIP levels compared with females, which is similar to the previous studies, AIP was not found to differ based on BMI, age, and disease subtypes in the present study. Even though this discrepancy could be explained by the difference in the study population and the study design, it seems apparent that the impact of AIP in health and diseases could be variable and should be better investigated.

### Study strengths and limitations

The most important strength of the present study was that it demonstrated, for the first time, that AIP at diagnosis is an independent predictor for CVA. However, several issues should be considered as limitations. First, the study was performed retrospectively and the clinical outcomes of the patients were identified by reviewing the hospital’s medical records. In addition, the optimal cut-off value of AIP in predicting CVA could not be defined by this study. Second, because only laboratory data at initial diagnosis was used to calculate AIP, it is unclear whether dynamic changes in AIP levels might be more relevant to the risk of CVAs. Third, the precise mechanism of how AIP is associated with increased CVA events could not be elucidated. Future prospective studies with a larger number of patients will help verify the results of this study and provide more information regarding the potential of AIP in predicting CVA in AAV.

## Conclusions

AIP in AAV patients was significantly higher than that in controls. Also, AIP ≥ 0.11 at diagnosis could predict CVA occurrence during follow-up. These results suggest that stringent surveillance is required in AAV patients with AIP ≥ 0.11 regarding the occurrence of CVA.

## Data Availability

The datasets used and/or analyzed during the current study are available from the corresponding author on reasonable request.

## Abbreviations

AAV: Antineutrophil cytoplasmic antibody-associated vasculitis
AIP: Atherogenic index of plasma
ANCA: Antineutrophil cytoplasmic antibody
BMI: Body mass index
BVAS: Birmingham vasculitis activity score
CAD: Coronary artery disease
CI: Confidence interval
CID: Chronic inflammatory disorder
CRP: C-reactive protein
CVA: Cerebrovascular accident
CVD: Cardiovascular disease
ESR: Erythrocyte sedimentation rate
FFS: Five-factor score
HDL: High-density lipoprotein
HR: Hazard ratio
IL: Interleukin
LDL: Low-density lipoprotein
MPA: Microscopic polyangiitis
TG: Triglyceride
TNF: Tumor necrosis factor
